# Developmental Abnormalities of the Pediatric Spine: A Review of the Correlation Between Ultrasound and MRI Findings

**DOI:** 10.7759/cureus.44580

**Published:** 2023-09-02

**Authors:** Parker Hunsaker, Kanika Gupta, Nolan Otto, Monica J Epelman, Tushar Chandra

**Affiliations:** 1 Diagnostic Radiology, University of Central Florida College of Medicine, Orlando, USA; 2 Diagnostic Radiology, University of Arizona, Tucson, USA; 3 Diagnostic Radiology, University of South Florida Health, Orlando, USA; 4 Diagnostic Radiology, Nicklaus Children's Hospital, Miami, USA; 5 Pediatric Radiology, Nemours Children's Hospital, Orlando, USA

**Keywords:** spinal developmental abnormalities, pediatric spine, spinal cord development, spinal ultrasound, spinal mri, spinal disraphysm

## Abstract

A broad spectrum of spinal pathologies can affect the pediatric population. Ultrasound (US) is the primary modality for pediatric spine assessment due to its widespread availability, non-requirement of sedation, and absence of ionizing radiation. Supplementing this, MRI offers an in-depth exploration of these conditions, aiding in preoperative strategizing. In this review, we examine the clinical indications, methodologies, and protocols for US and MRI scans of the pediatric spine. Additionally, we illustrate normal pediatric spinal anatomy, highlighting several examples of normal variants that are often misinterpreted. Through a series of case-based illustrations, we offer a comprehensive overview of various pathological conditions such as tethered cord, spinal dysraphism, spinal lipoma, diastematomyelia, and dermal sinus tract, among others. Furthermore, we explore the correlation between US and MRI findings for these lesions, employing real-world cases to enhance our understanding of this topic.

## Introduction and background

Spinal cord lesions are not an uncommon occurrence in the pediatric population, and the failure to recognize and treat them can result in permanent neurologic deficits [1,2]. While open spinal lesions (characterized by a defect in the overlying skin) are easily recognizable, those that are closed can be easily overlooked if careful attention is not paid to the classical signs. Commonly asymptomatic, 50-80% of closed spinal lesions are associated with cutaneous stigmata that are present from birth, as delineated in Table 1 [1,3]. The presence of these signs typically warrants further imaging to screen for spinal pathology.

**Table 1:** Cutaneous markers of spinal dysraphism

Markers
Asymmetric gluteal cleft
Dermal sinus tract
Hypertrichosis
Hemangiomas
Deep dimples and pits
Midline mass
Pigmented nevus
Port wine stain
Sacral dimples
Skin tags
Subcutaneous lipoma
Telangiectasias

Ultrasound (US) is the first-line imaging modality to screen for pediatric spinal lesions [4]. It is cost-effective, portable, non-invasive, and does not use radiation. Moreover, sedation of the patient is not required, which avoids the risks of anesthesia, such as causing harm to the pediatric nervous system [5]. Indications for pediatric spinal US, other than the cutaneous stigmata, are listed in Table 2.

**Table 2:** Indications for pediatric spinal ultrasound as proposed by the American Institute of Ultrasound in Medicine (AIUM)

Indications
1. Cutaneous stigmata known to be associated with spinal dysraphism (see Table 1)
2. Spectrum of caudal regression syndrome
3. Evaluation of suspected defects such as cord tethering, diastematomyelia, hydromyelia, and syringomyelia
4. Detection of a sequel of injury (e.g., subdural hematoma)
5. Visualization of fluid with characteristics of blood products within the spinal canal in patients with intracranial hemorrhage
6. Guidance for lumbar puncture
7. Postoperative assessment for cord retethering

While US serves as an excellent screening tool for closed spinal lesions in pediatric patients, in many cases, it is difficult to establish a definitive diagnosis based on US findings alone. In such cases, MRI is usually performed as a follow-up study for further characterization and surgical planning. MRI often serves as a superior diagnostic tool due to its multiplanar capability, excellent soft tissue resolution, and enhanced soft tissue contrast.

In this review, we aim to outline the commonly used protocols for US and MRI of pediatric spinal lesions. We also intend to highlight the correlation and differences between US and MRI findings in terms of several pediatric spinal cord lesions.

## Review

US technique

US of the lumbar spine in pediatric patients is performed with high-frequency linear transducers (7-12 MHz) in longitudinal and transverse planes [6,7]. Infants who are fed just prior to the exam tend to be calmer, facilitating the capture of high-quality images. It is best for the infants to be laid prone with a pillow placed underneath them in a way that slightly elevates the upper body. This position allows the spinal fluid to accumulate in the lower thecal sac, widening the interlaminar spaces and thus forming a larger acoustic window [3,8,9].

Various US modes may aid in the diagnosis of closed spinal lesions. For example, extended field-of-view (panoramic) imaging can be used to capture the spine from the coccyx to T12, making it a useful tool for counting vertebral levels. Cine imaging is useful in depicting anatomy as well as the movement of the spinal cord and nerve roots that occur with spinal CSF pulsations. M-mode is helpful in documenting these movements [3,6].

US Normal Anatomy

**Figure 1:**
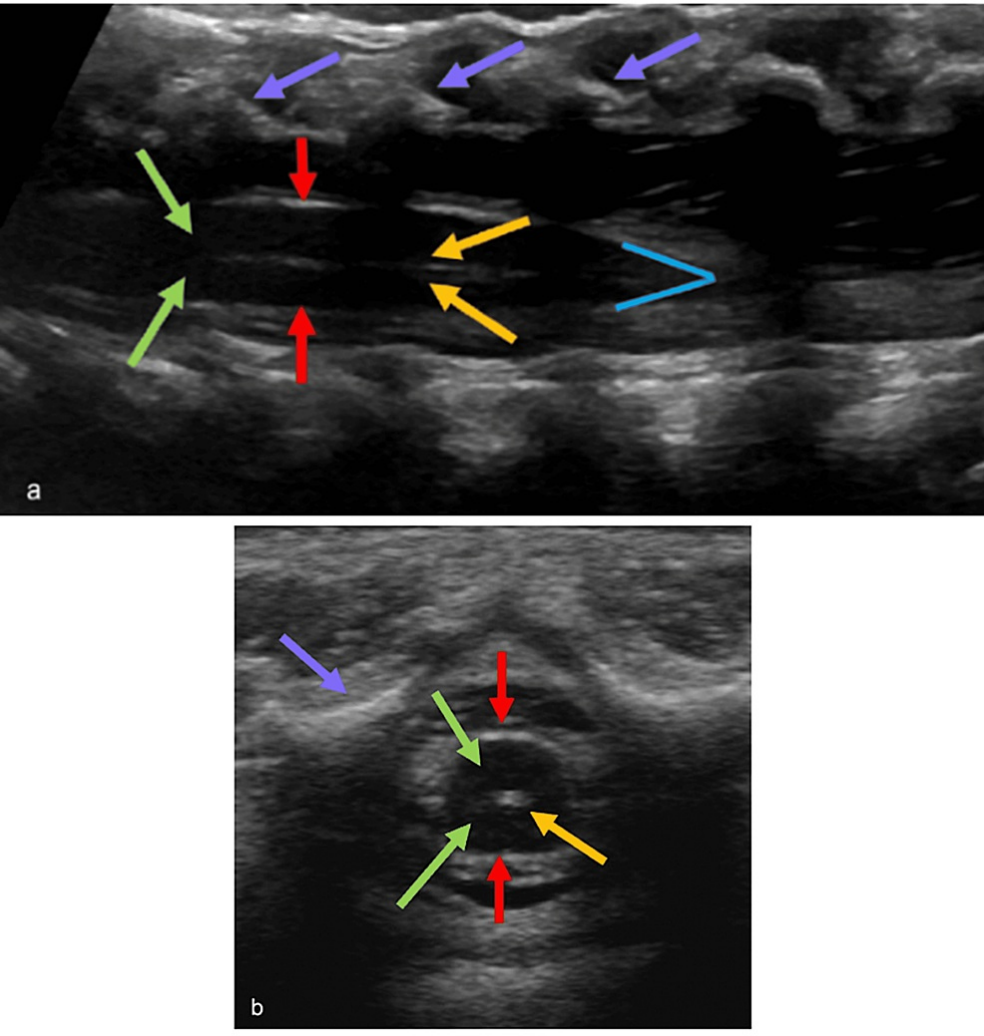
Normal cord anatomy on ultrasound Longitudinal (a) and transverse (b) ultrasound images demonstrating normal cord anatomy. The vertebrae appear as echogenic structures anterior to the cord (purple arrows). The spinal cord is a tubular hypoechoic structure with hyperechoic walls (red arrows). The central echo complex is demonstrated by an echogenic structure located centrally within the spinal cord (yellow arrows). Hypoechoic subarachnoid space surrounds the cord (green arrows). The conus medullaris appears as a V-shaped tapering of hyperechoic walls at the end of the cord (blue V) Radiological images obtained by the authors

To accurately recognize and diagnose closed spinal lesions with sonography, one must first be able to identify the normal elements of the pediatric spine, particularly in the lumbosacral area. The first echogenic line seen proximal to the probe is the dermis. Immediately beneath is a hypoechoic layer of subcutaneous fat with echogenic striations coursing through it, representing connective tissue (Figure 1) [10]. 

The spinous processes are found below the subcutaneous fat and dorsal to the thecal sac. The ossified portions appear echogenic, but these structures are predominantly hypoechoic, as the pediatric spine is mostly composed of cartilage at birth. The vertebral bodies can be seen ventral to the spinal cord and appear echogenic with posterior acoustic shadowing.

The thecal sac and its contents are located between the spinous processes and vertebral bodies. The dura mater, which makes up the walls of the thecal sac, appears as echogenic parallel lines on the longitudinal axis and a circular perimeter on the transverse axis. Directly beneath the dura mater is the subarachnoid space, filled with anechoic CSF that surrounds the spinal cord. The nerve roots of the spinal cord can be seen within this space as echogenic lines, coursing posteriorly on longitudinal images and laterally on transverse images.

The spinal cord itself is a tubular structure with similar echogenicity to that of the CSF. However, its interface with the subarachnoid space is echogenic. The central canal of the spinal cord forms an echogenic structure known as the central echo complex, which forms a parallel line longitudinally and a dot transversely. The echogenicity of this complex is produced by the interface of the two margins of the central canal. The conus medullaris is located at the most caudal portion of the spinal cord, where it smoothly tapers together to form a cone-like structure. It is surrounded by the nerve roots of the cauda equina.

The filum terminale extends distally from the conus medullaris toward the caudal end of the spinal canal. It is an echogenic structure, measuring 1-2 mm in thickness, that has a hypoechoic portion at its center [8,11]. It is important to measure the size of the filum terminale, as excessive thickness may be an indicator of pathology. The filum terminale and the nerve roots of the conus medullaris should not attach to each other or to the walls of the thecal sac. These structures are determined to be floating freely if their motion is observed in coordination with CSF pulsations on real-time sonography or cine clips. M-mode may be useful in documenting whether this motion is present or not [3,6]. 

Assigning Vertebral Level

One of the most crucial steps in reading a pediatric spinal US image is identifying the exact location of the conus medullaris within the spinal canal. A conus located below the L3 vertebral body may be a sign of a low-lying spinal cord or tethered cord syndrome [12,13]. Determining the location of the conus medullaris requires the correct enumeration of vertebral levels, for which there are several reliable methods.

**Figure 2:**
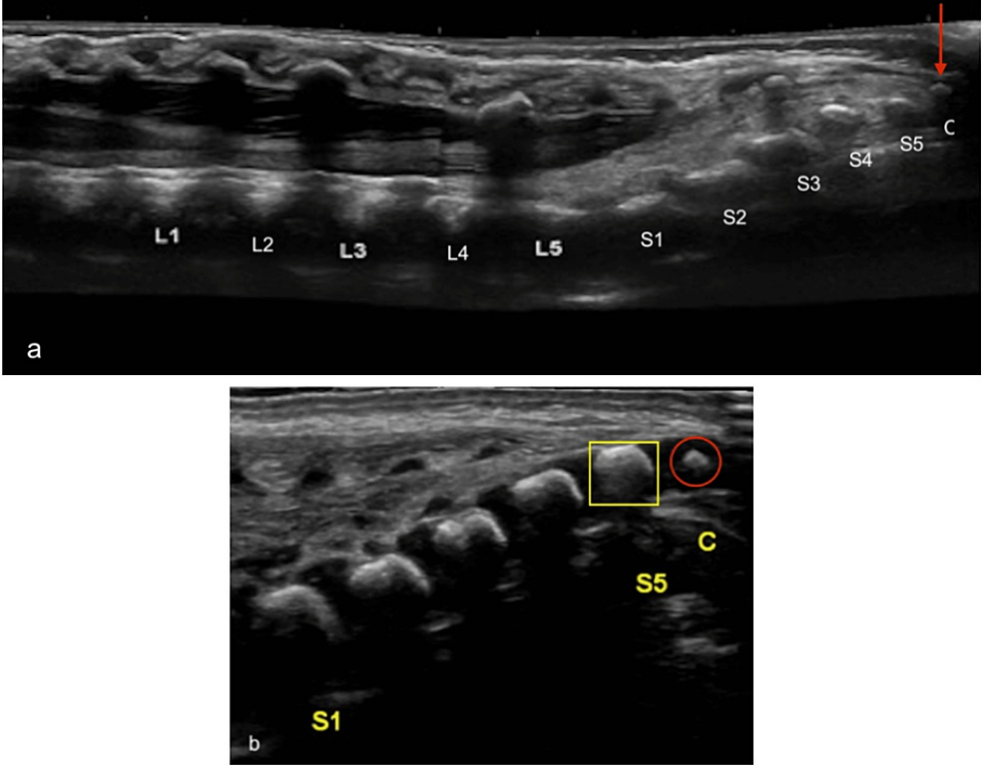
Coccyx appearance and location (a) Longitudinal ultrasound image demonstrating the coccyx (red arrow) as the last echogenic structure at the distal end of the spine. (b) The ossification center of the coccyx has a rounded configuration (red circle), as opposed to the squarish appearance (yellow square) of sacral ossification centers Radiological images obtained by the authors

The first method for assigning the vertebral level involves counting each vertebral body superiorly from the coccyx. The coccyx is found by locating the last echogenic structure at the distal end of the spine (Figure 2). The coccyx may also be differentiated by the rounded configuration of its ossification center, as opposed to the more squared appearance of the other vertebral bodies [3].

**Figure 3:**
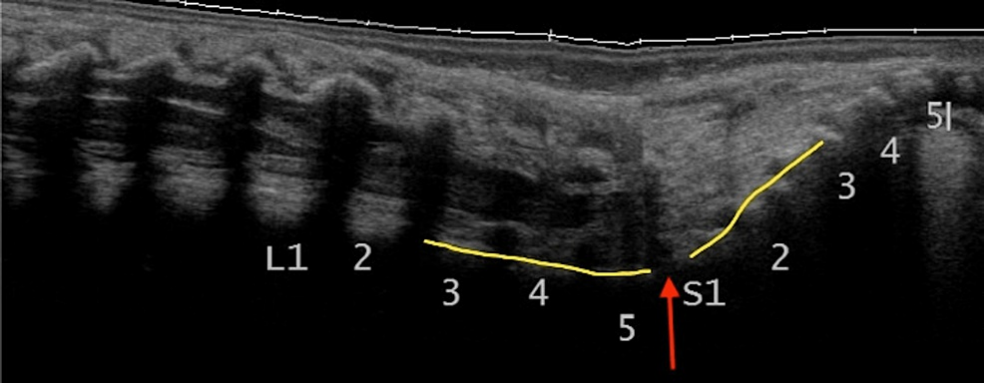
Lumbosacral junction Longitudinal ultrasound image demonstrating the lumbosacral junction (red arrow) as an abrupt change in angulation between L5 and S1 Radiological image obtained by the authors

Another reliable method is to define the lumbosacral junction. This junction is distinguished by an abrupt change in angulation between the lumbar and sacral vertebrae, with the latter tilting posteriorly towards the surface of the skin [14,15]. Therefore, identification of the junction between L5 and S1 provides orientation within the spine, facilitating vertebral counting (Figure 3).

**Figure 4:**
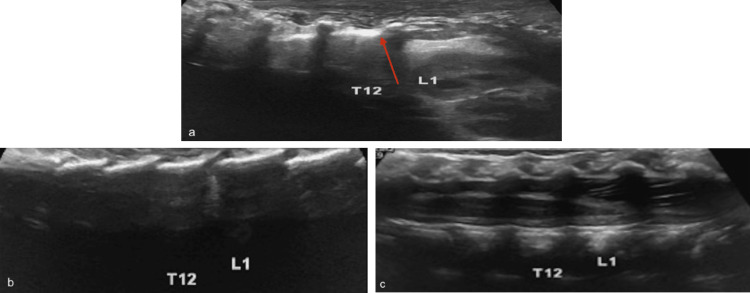
Rib shadow Lateral (a), paramedian (b), and midline (c) longitudinal ultrasound images demonstrating the last rib shadow (red arrow) on the T12 vertebra. Moving the probe medially while following the T12 vertebral shadow helps in assigning the levels of T12 and L1 vertebrae Radiological images obtained by the authors

While not as reliable as the other methods, it may be useful to confirm correct counting by identifying the last rib-bearing vertebra as well as the end of the thecal sac (Figure 4). For most people, the last rib is connected to T12, and the thecal sac ends at S2. This allows us to count caudally and cephalad, respectively, from these sites to determine the location of the conus medullaris [3,16]. However, the position of these markers is not the same in every individual, and hence it is important to always use a combination of methods to ensure the accuracy of vertebral labeling.

MRI protocol

Spinal MRI can be performed with either a 1.5T or 3T scanner [17]. All spinal imaging with MR should utilize sagittal T1 and T2-weighted sequences with a field of view of 200-250 mm and a slice thickness of 3-4 mm [17-19]. Short-tau inversion recovery (STIR) is also useful in amplifying the signal of abnormalities with subtle signal intensity in the spinal cord as well as the myofascial planes. Contrast is not typically necessary in spinal MRI unless in the case of an inflammatory or oncologic disease. In these cases, intravenous gadolinium administration followed by a post-contrast T1 scan may be useful [18].

Embryology and development of spine

To learn about normal and pathological variants of the spinal cord, it is important to have a foundational understanding of spinal cord development. The spinal cord develops in two stages: primary neurulation and secondary neurulation. 

**Figure 5:**
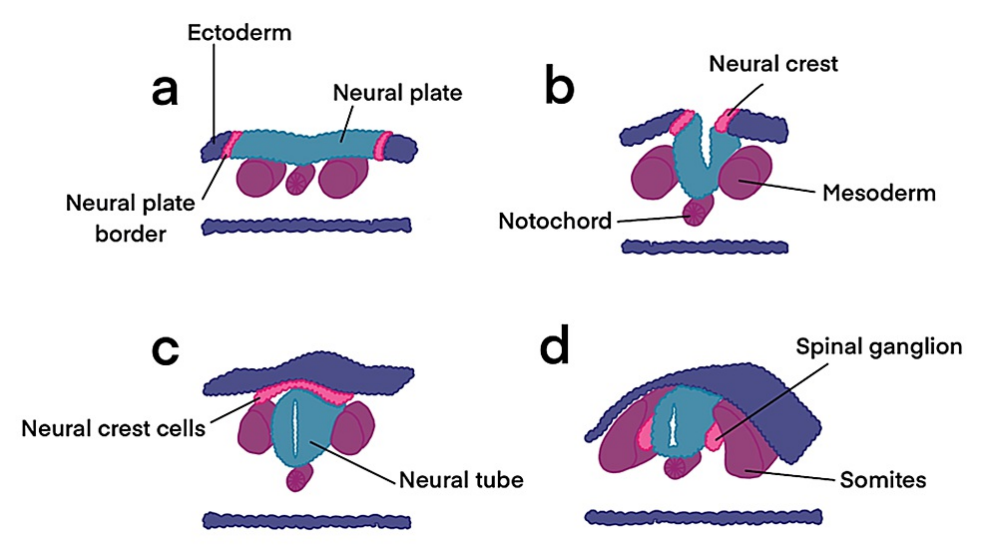
Primary neurulation Illustration demonstrating the process of primary neurulation, which occurs during weeks 3-4 of embryogenesis. (a) The neuroectoderm differentiates from the cutaneous ectoderm and thickens to form the neural plate. (b) A groove is formed as the neural plate begins to fold dorsally, with the two ends eventually joining at the neural plate borders, which are now referred to as the neural crest. (c) As the neural tube closes, the neural crest disconnects from the epidermis. (d) Migration of the neural crest cells forms the dorsal root ganglion and other structures. The notochord will form the vertebral column Adapted from Anatomy and Physiology (Figure 28.13) with permission from OpenStax under CC license

Primary neurulation is the process by which the neural tube forms. It occurs in the third and fourth weeks of embryogenesis after the endoderm, mesoderm, and ectoderm germ layers have formed. In this stage, the notochord sends signals to a portion of the overlying ectoderm, causing it to form the neural plate [20]. The neural plate then folds inward at its center until its two ends meet, separating itself from the cutaneous ectoderm and forming the neural tube (Figure 5) [21].

Secondary neurulation occurs in the fifth week of embryogenesis. It begins with the formation of a mass of cells at the caudal end of the embryo. The cells in this mass come together and canalize before fusing with the distal end of the neural tube [20]. The fused caudal cell mass and neural tube then undergo a process called retrogressive differentiation, in which they form the conus medullaris, ventriculus terminalis, and filum terminale [22].

Normal variants

It is important to identify anatomical variants and differentiate these from pathologies that would require further investigation. This protects the child from unnecessary medical risks, including potential sedation, exposure to ionizing radiation, or surgical morbidity.

Transient Dilatation of the Central Canal

**Figure 6:**
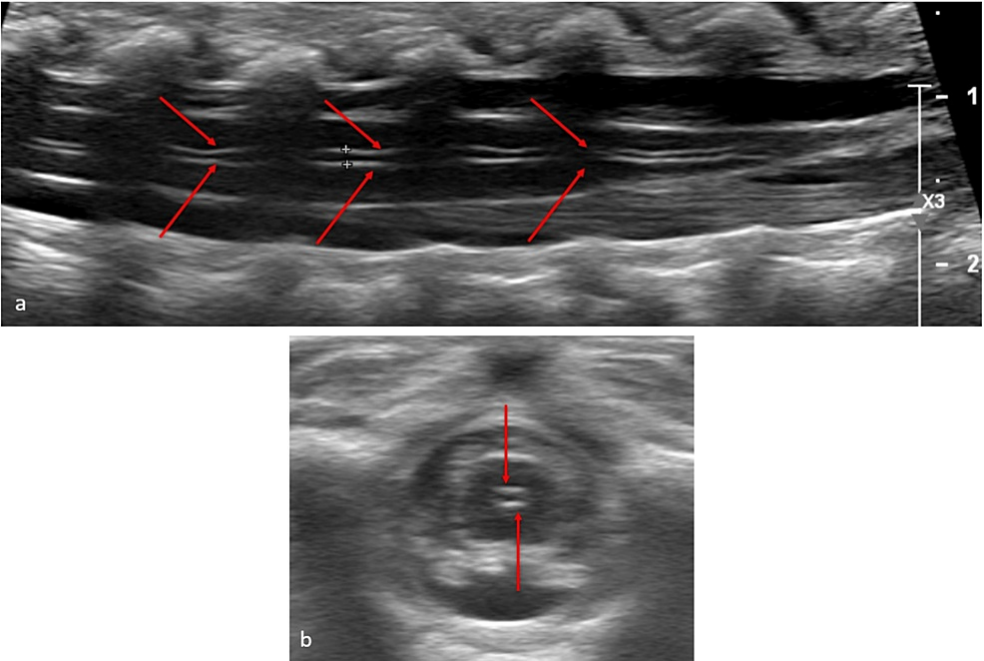
Dilatation of the central canal Longitudinal (a) and transverse (b) ultrasound views demonstrating the splitting of the central echo complex (red arrows) Radiological images obtained by the authors

Mild, diffuse dilatation of the central canal may be observed within the first few weeks of life before spontaneously resolving. On a single exam, this may be difficult to differentiate from syringohydromyelia, which would be expected to be thicker and persist into childhood [23]. Normally, the central canal is represented by a single echogenic line centrally within the spinal cord, produced by reflected echoes at the transition from the anterior commissure to the median anterior fissure of the spinal cord [24]. Splitting of this central echo complex by an anechoic strip indicates a dilated central canal containing CSF (Figure 6). Mild, diffuse dilatation of the central canal occurring in isolation without associated anomalies on sonography may not require further examination with MRI; follow-up imaging with ultrasound after the first few months of life is a reasonable option to demonstrate resolution and rule out syrinx.

Ventriculus Terminalis

The process of canalization and retrograde differentiation during secondary neurulation yields the terminal ventricle within the conus medullaris [20]. The ventriculus terminalis is an ependyma-lined structure continuous with the central canal, which usually regresses within the first few years of life. 

**Figure 7:**
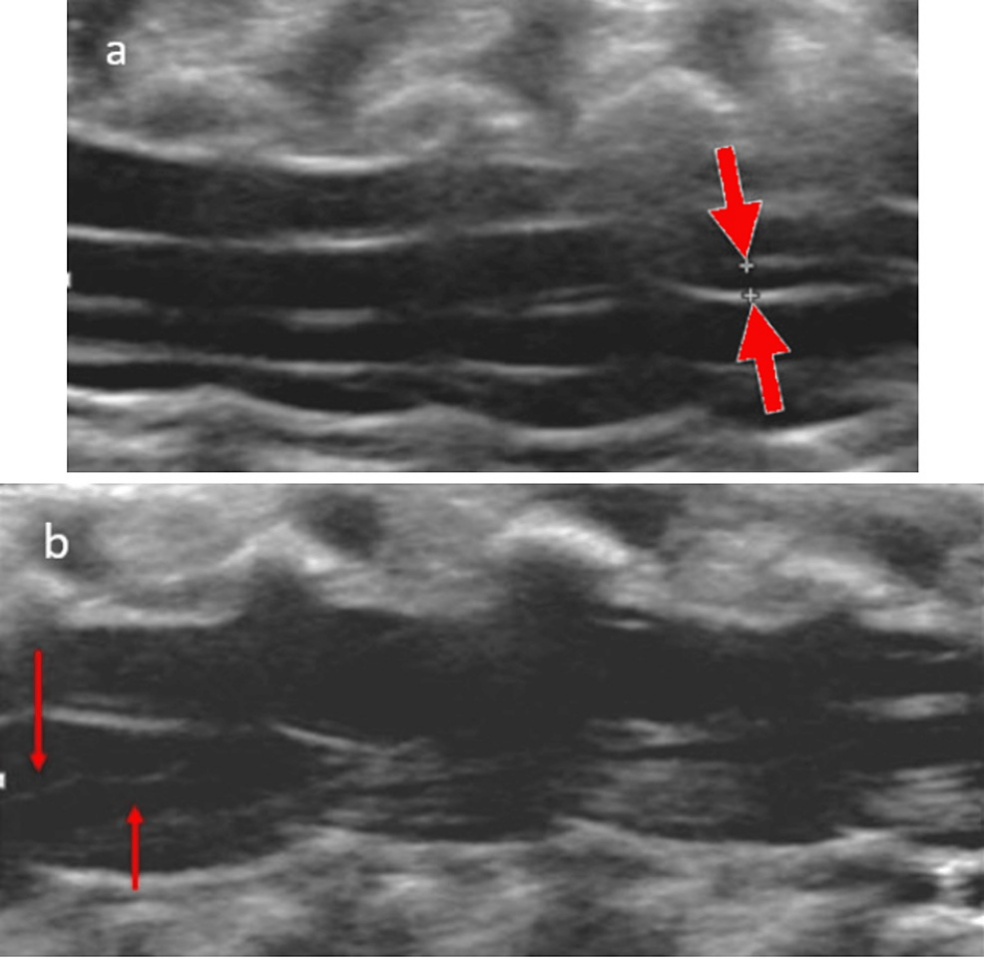
Ventriculus terminalis (a) Longitudinal ultrasound image demonstrating cystic dilatation within the conus medullaris continuous with the central canal (red arrows). (b) Follow-up ultrasound at 6 weeks of age demonstrates resolution Radiological images obtained by the authors

Ultrasound shows an anechoic cystic dilation continuous with the central canal within a well-positioned and morphologically normal conus (Figure 7). The borders are hyperechoic and measure 2-4 mm in the axial dimension and 8-10 mm in the longitudinal dimension [25,26]. MRI shows T2-hyperintense cystic dilation at the conus without surrounding cord edema. On T1-weighted sequences, the cystic space is non-enhancing and hypointense consistent with CSF.

Prominent, Thickened Filum Terminale and Central Echo Complex

Increased echogenicity or diameter of the filum terminale may raise concerns for a potential tethered cord. The filum may be considered thickened when its diameter exceeds 2 mm. The filum may be considered “prominent” when it is hyperechoic compared to the surrounding spinal nerves of the cauda equina [27]. A linear echogenic structure continuing from the central echo complex of the spinal cord may be observed and is consistent with a continuation of the central canal [27]. These may be differentiated from a tethered cord by observing the midline position of the filum within the spinal canal, normal positioning of the conus above the L2-L3 disc space, and absence of neurological deficits [11,28]. For measuring purposes, the transverse view may be best in differentiating a filum of normal echogenicity from the surrounding nerve roots [27]. MRI is able to differentiate a widened fibrous filum from lipomatous infiltrations due to a lack of T1 hyperintensity in the former [29].

Pseudo-Sinus Tract

A cord-like fibrous structure may extend from the coccyx to the dermis. The thecal sac normally ends around S2 and, therefore, will have no communication with a pseudo-sinus tract terminating at the coccyx [28]. US may show a midline hypoechoic curvilinear or linear tract extending posteriorly from the coccyx to the skin. On MRI, the tract is T1- and T2-hypointense without enhancement or restricted diffusion. There is no evidence of T1-hyperintense dermoids or T2-intense CSF along the length of the tract.

Overt spinal dysraphisms

Overt spinal dysraphisms result from deranged primary neurulation. Improper separation of the cutaneous and neural ectoderms results in exposed neural elements [15]. The incomplete closure of the posterior vertebral arches is referred to as spinal bifida [1]. Bifid posterior arches allow an opening for the contents of the spinal canal to protrude.

Myelocele and Myelomeningocele

Overt spinal dysraphisms include myelocele and myelomeningocele. In either case, the neural placode (remnant of undeveloped neural ectoderm) is continuous with the skin. In myelocele, the placode is level with the surrounding skin. In myelomeningocele (i.e., spina bifida cystica), the placode is elevated by meningeal expansion through the vertebral defect. Because ultrasound is contraindicated for direct investigation of the open spinal defect, MRI is necessary to characterize the dysraphism as well as associated cerebral and cerebellar malformations. T2-weighted sequences are especially useful for demonstrating the expansion of the meningeal spaces [30].

Occult spinal dysraphisms

Closed spinal dysraphisms include a variety of consequences of neural tube maldevelopment, including mesodermal, vertebral, meningeal, spinal cord, and filum. They are less obvious due to the intact overlying skin but may have overlying cutaneous markers (see indications for spinal ultrasound, Table 1).

Meningocele and Myelocystocele

Meningoceles are caused by CSF-filled meningeal cysts herniating through vertebral defects. These may herniate posteriorly or anteriorly and are more common in the lumbosacral region but may also occur in the thoracic or cervical spine [31]. In myelocystocele, there is both a meningocele as well as a dilated central canal, and both may protrude dorsally through a bifid spine into the subcutaneous tissues.

Ultrasound is important for prenatal diagnosis of meningoceles and myelocystoceles [32,33]. Postnatally, sonography shows a cystic mass(es) extending past vertebral arch defects [34]. No neural tissue is demonstrated in meningocele [35]. Associated tethered cord may be seen [34,35]. In meningoceles, continuity between the central canal and subarachnoid spaces may be illustrated with the aid of sonography [35].

In myelocystocele, the interface of the terminal syrinx and meningocele is often complex. Either or both may herniate into the subcutaneous tissues, either may be the larger defect, or they may wrap around each other [31,33,34,36]. While sonography alone may identify the presence of the terminal syrinx, meningocele, subcutaneous cystic structure(s), and vertebral defects, it may be insufficient for a complete description. MRI provides data better suited for a more precise anatomical description and can detect abnormal flow dynamics [36].

MRI reveals changes in fluid dynamics not previously described on ultrasound. Flow voids and dephasing artifacts are revealed on T2-weighted imaging, representing turbulent CSF flow. The turbulent flow may be present within either or both of the terminal syrinx (especially at the point where the central canal opens into the terminal syrinx) or the meningocele [31,36].

Spinal Lipomas

**Figure 8:**
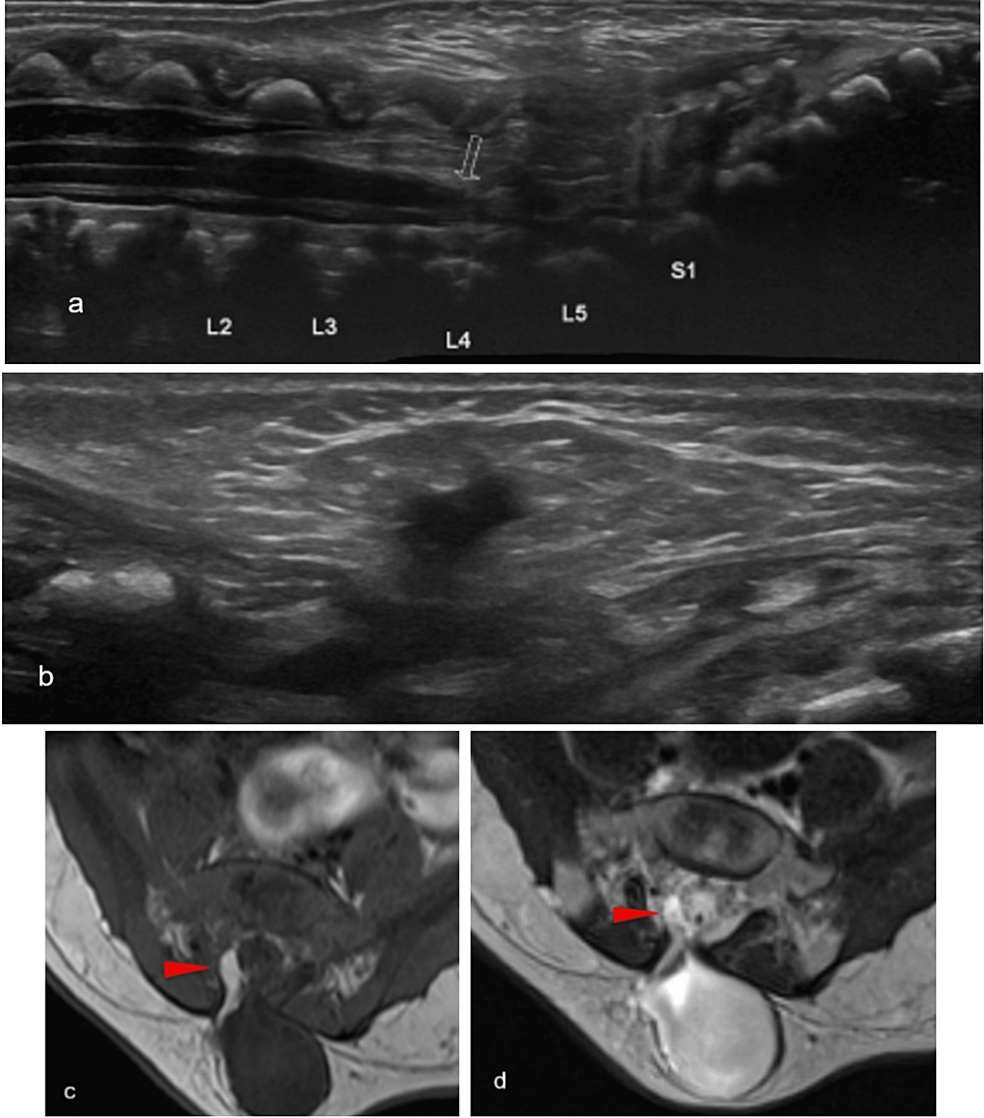
Spinal lipoma Longitudinal (a) and transverse (b) ultrasound images showing the cystic structure in soft tissues overlying L4-5. T1 (c) and T2 (d) MR images in the axial plane depicting a T1 hyperintense component, in keeping with fat Radiological images obtained by the authors

Spinal lipomas result from premature separation of the cutaneous and neural ectoderm and resultant infiltration of mesenchymal tissue into the developing neural tube [20]. Lipomyelocele and lipomyelomeningocele are differentiated by the location of the lipoma-placode mass; it is contained in the spinal canal in lipomyelocele, while it extends outside the spinal canal in lipomyelomeningocele. Ultrasound demonstrates echogenic subcutaneous lipoma and infiltrating fatty tissue interfacing with the spinal cord and cauda equina. Spinal lipomas are commonly associated with low-lying conus and tethered cord. Cord tethering is implied by M-mode, especially by the absence of nerve root pulsations [15,35]. MRI provides a higher resolution of the infiltrating subcutaneous fat, placode-lipoma interface, and position relative to the spinal canal (Figure 8). In both lesions, T1-weighted sequences demonstrate hyperintense subcutaneous mass. T1-bright fat adjacent to relatively hypointense neural tissue represents the placode-lipoma interface [15,31].

Fibrolipoma of the filum terminale typically presents on ultrasound as a thickened, echogenic filum. Fibrolipoma may be difficult to differentiate from prominent or thickened filum on ultrasound. MRI demonstrates T1-bright fatty tissue, which is suppressed on fat-saturating sequences within an otherwise hypointense filum [15,35].

Dorsal Dermal Sinus

A dorsal dermal sinus connotes the communication between the skin and subarachnoid space via an epithelial-lined tract. Dorsal dermal sinus may be associated with other spinal dysraphisms, split cord malformations, tight filum terminale, or low-lying cords [20].

**Figure 9:**
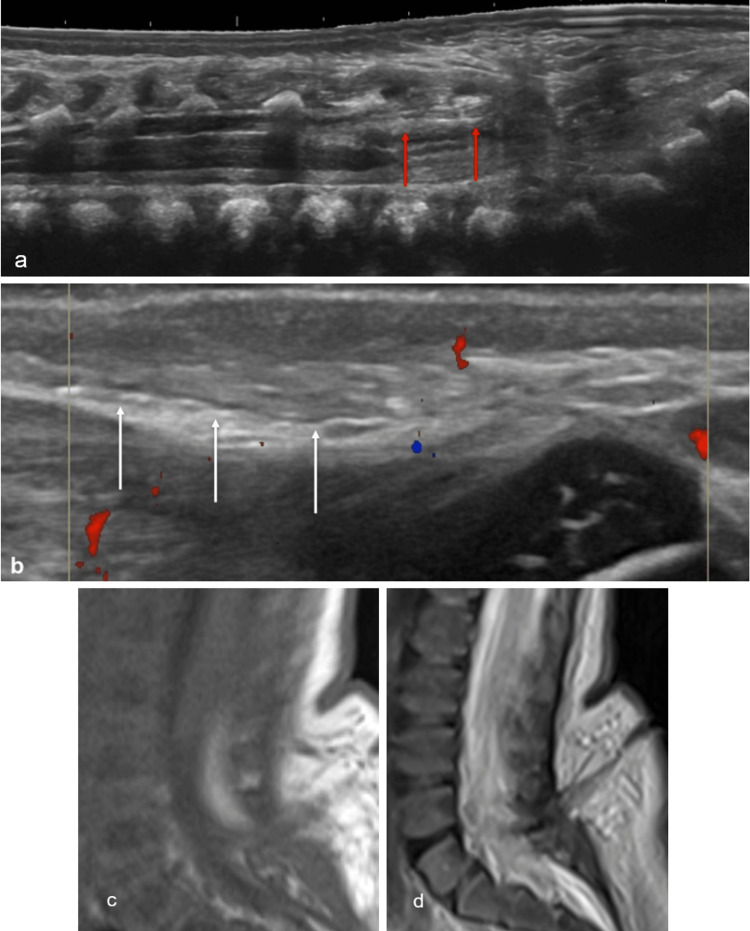
Dorsal dermal sinus Longitudinal (a, b) ultrasound images demonstrate a low-lying conus with an echogenic structure dorsally within the spinal canal below the level of the conus (red arrow). Also noted is a linear tract extending into the subcutaneous plane (white arrow). Sagittal T1 (c) and T2 (d) MR images confirm the low-lying conus at L4 and the hypoechoic tract connecting the skin to the distal thecal tissues at L5-S1, at the site of bulging of soft tissues, compatible with the patient’s sacral dimple Radiological images obtained by the authors

The investigation starts with an ultrasound. In the skin, the tract is hypoechoic adjacent to relatively hyperechoic subcutaneous fat. In the subarachnoid space, the tract is echogenic within anechoic CSF. MRI is used to characterize any associated anomalies. The tract is T1- and T2-hypointense from the skin to the dura [20]. If present, a dermoid or epidermoid mass is T2-hyperintense, while lipomas are T1-hyperintense (Figure 9).

Split Cord Malformations

The spinal cord may be split into hemicords at varying positions and with varying degrees of symmetry, but always with their own central canal. Diastematomyelia is commonly used synonymously [37,38].

Type I refers to hemicords with their own dural sac and subarachnoid space and are separated along the midline by a rigid osseous or cartilaginous septum. In type II, the hemicords share a single dural sac and subarachnoid space, with a fibrous non-rigid intervening septum. Hemicords often have associated anomalies, including hydromyelia, other spinal dysraphisms, or kyphoscoliosis, but are most often associated with a tethered cord [39].

On ultrasound, the separation between the hemicords may be difficult to visualize in the longitudinal plane. In the axial plane, the hemicords, as well as a hypoechoic intervening septum, may be visualized. A central echogenic complex representing the central canal is found within each hemicord.

**Figure 10:**
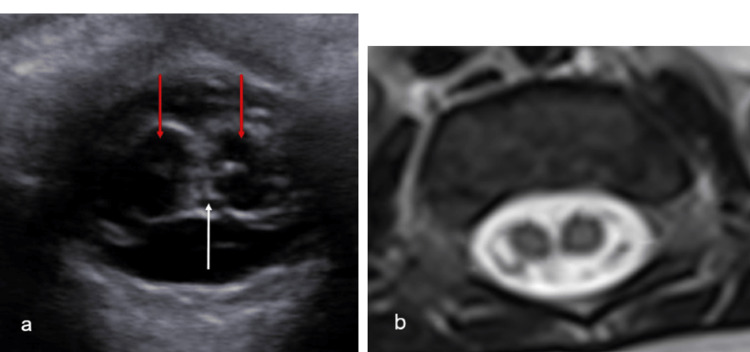
Hemicords (a) Transverse ultrasound image demonstrating two hemicords (red arrows), separated by a thin septum (white arrow). (b) Axial T2-weighted MR image of the same patient confirms the findings Radiological images obtained by the authors

Split cord malformations are best shown on MRI in the axial and coronal planes, as viewing the septum and dural tubes may be difficult in the sagittal view [40]. T2-weighted sequences show distinct hemicords and may possibly show individual T2-hyperintense central canals (Figure 10). MRI is sensitive to associated anomalies.

Tethered and Low-Lying Cord

**Figure 11:**
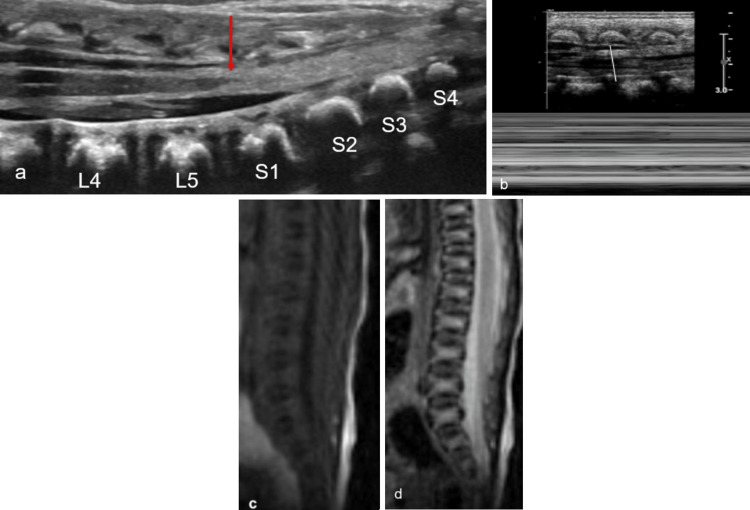
Tethered cord (a) Longitudinal ultrasound image demonstrating low-lying cord at the level of L5-S1 with echogenic, thickened filum >2 mm, and dorsal position of the conus and filum (red arrow). (b) Ultrasound M-mode illustrating the absence of pulsations of the cauda equina. Sagittal T1 (c) and T2 (d) MR images confirm the low-lying cord without any other associated abnormality Radiological images obtained by the authors

A spinal cord can become “tethered” when, due to a variety of etiologies, the filum restricts the dependent range of motion. Ultrasound should be the first modality of imaging. Sonography shows a low-lying conus below the L2/L3 vertebral levels [34]. Limited dependent motion with patient positioning may be apparent such as with decubitus positioning with sonogram or prone MRI. The motion of the conus and cauda equina may be demonstrated by cine images and M-mode (Figure 11). Sonographic re-evaluation following detethering surgery is useful for detecting cord retethering.

Tethered cord is the most common finding associated with split cord malformations, which have been described above [35].

Caudal Regression Syndrome

Errors in the development of the caudal cell mass and integration with the neural tube during secondary neurulation result in malformations of the lumbosacral spine and conus medullaris and may disrupt the lower extremities. Their etiology is unclear but could be associated with maternal diabetes and genetic variations in retinoic acid metabolism [38,41].

**Figure 12:**
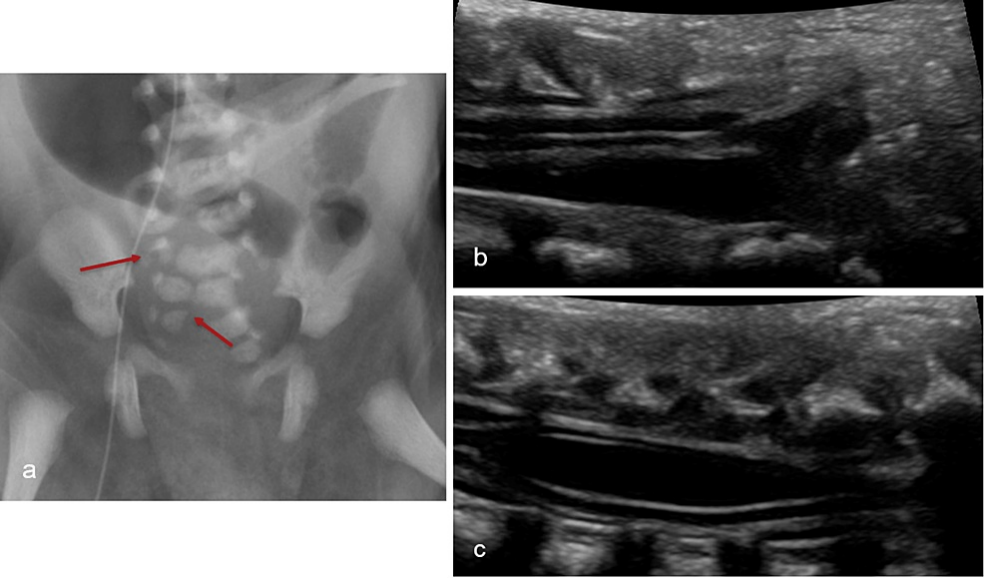
Caudal regression syndrome (a) AP radiograph of the pelvis depicting dysmorphic sacrum with right-sided hypoplasia/agenesis of the distal sacrum (red arrows). (b, c) Longitudinal ultrasound images illustrating low-lying dysmorphic conus with syringohydromyelia in keeping with type 2 caudal regression syndrome Radiological images obtained by the authors

Caudal regression syndromes are classified into two groups. In group one, there is more severe caudal dysgenesis, which is thought to be due to a decreased number of anterior horn cells. There is an associated high-lying (above L1 level) club- or wedge-shaped conus. In group two, there is less severe caudal dysgenesis, but the cord is tethered and may have associated spinal lipoma (Figure 12) [42]. Sacral spinal pathology is implicated in several congenital developmental syndromes, including Currarino syndrome, VACTERL (Vertebral defects, Anal atresia, Cardiac defects, Tracheoesophageal fistula, Renal anomalies, and Limb abnormalities) association, OEIS (Omphalocele, Exstrophy of the cloaca, Imperforate anus, and Spine abnormalities) complex, and limb-body wall defect (LBWD).

## Conclusions

US is the first-line modality for imaging the pediatric spine during the first few months of life due to incomplete ossification of the posterior spine. Screening for spinal lesions in this age group is recommended for those with any of the indications listed in Table 2 above. Despite the superior imaging quality obtained with MRI, it is not ideal for screening due to long scan times, high cost, and the need for sedation in some cases. However, when an ultrasound is positive for an abnormality that is not a normal variant, an MRI is typically indicated to further characterize the abnormality. MRI is beneficial in these cases due to its multiplanar capability, excellent soft tissue resolution, lack of ionizing radiation, and its utility in pre-surgical planning.
